# Biological intensity-modulated radiotherapy plus neoadjuvant chemotherapy for multiple peritoneal metastases of ovarian cancer: A case report

**DOI:** 10.3892/ol.2014.2820

**Published:** 2014-12-22

**Authors:** SHIGAO HUANG, YAZHENG DANG, FUJUN LI, WEI WEI, YUXIN MA, SONG QIAO, QIANYUN WANG

**Affiliations:** Department of Oncology, Chinese People’s Liberation Army 323 Hospital, Xi’an, Shaanxi 710054, P.R. China

**Keywords:** peritoneal multiple metastasis, ovarian cancer, radiotherapy, neoadjuvant chemotherapy

## Abstract

The current study presents the case of a 68-year-old female patient who received biological intensity-modulated radiotherapy (BIMRT) and neoadjuvant chemotherapy for multiple peritoneal metastases of ovarian cancer. The International Federation of Gynecology and Obstetrics disease stage was IIIc. In addition, the patient presented with urination and defecation difficulties. The result of tumor marker detection showed a carcinoembryonic antigen level of 348.2ng/ml, a cancer antigen 125 level of 2,091 U/ml and a cancer antigen 19-9 level of 113 U/ml. Computed tomography (CT) indicated and ovarian cystic or solid package, enlargement of multiple abdominal and retroperitoneal lymph nodes and abdominal cavity effusion. Positron emission tomography/CT indicated multiple internal organ metastases. The center of the ovarian cystic or solid package was considered to be a malignant tumor. A large amount of ascites were detected, as well as abdominal and retroperitoneal lymph node metastasis. The patient was treated with BIMRT at a total dose of 48 Gy, administered as a single 4.0-Gy dose 12 times. In addition, 100 mg cisplatin was administered as a peritoneal perfusion, followed by two cycles of 180 mg Taxol and 100 mg cisplatin. Furthermore, the enlargement of the lymph nodes was reduced and the tumor in the region of the ovary had decreased in size by 90%. The ascites had disappeared and the abdominal pain was greatly improved. At the time of writing this manuscript, the patient was well and without relapse. Therefore, modern radiotherapy techniques, such as BIMRT, may be considered as a beneficial treatment option for ovarian cancer patients with multiple peritoneal metastases in whom surgery is not suitable.

## Introduction

As the fifth most common cause of cancer-related mortality, ovarian cancer accounts for >50% of all mortalities associated with gynecological cancer (1). With an incidence of 35–78%, nodal metastases occur frequently, particularly in advanced-stage ovarian tumors (stages III-IV) (2,3).

Primary debulking surgery (PDS) is the current standard treatment for advanced ovarian cancer, followed by post-surgical chemotherapy (4). An improved prognosis may be expected in cases where optimal debulking (residual disease, <1 cm) can be achieved (5). Neoadjuvant chemotherapy (NAC) has been recognized as an alternative treatment to PDS for patients with a poor performance status or apparently unresectable bulky tumors (6). NAC is expected to become a standard treatment or one of the effective treatment options for advanced ovarian cancer (7) as other phase III studies (8,9) begin to produce similar positive results. Radiotherapy may be an effective treatment modality, even in the setting of otherwise chemotherapy refractory disease (10). Tactics for the consolidation of a complete response following chemotherapy remain of great interest, but future studies are required to determine which consolidation treatment is optimal for advanced ovarian cancer (11).

A previous study has indicated that the majority of females with advanced ovarian cancer, in whom tumor control was achieved, will go on to develop recurrent disease (12). Combined positron emission tomography/computed tomography (PET/CT) is particularly useful for differentiating between ovarian cancer and benign disease, and for locating distant metastases. Borderline tumors may be interpreted as benign on PET/CT (13). In the present study, PET/CT technology was used to guide the use of the radiotherapy. The study was approved by the Ethics Committee of the People’s Liberation Army (PLA) 323 Hospital (Xi’an, China) and the patient provided written informed consent.

## Case report

**Figure 1:**
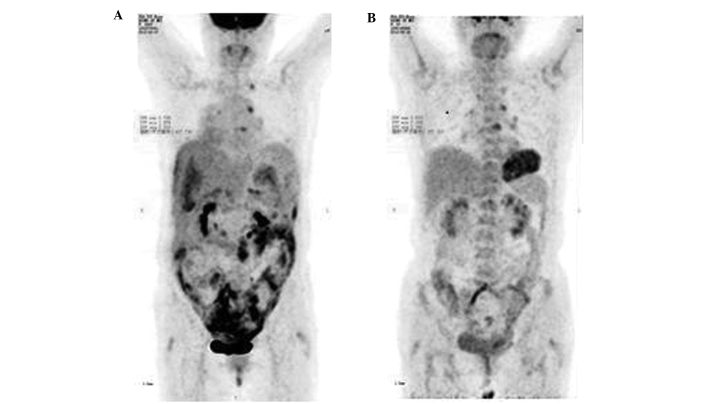
(A) PET revealing abnormal ^18^F-fluorodeoxyglucose uptake in the ovary and enterocoelia, indicating a malignant ovarian tumor. (B) PET images obtained at one year post-radiotherapy treatment. PET, positron emission tomography.

**Figure 2:**
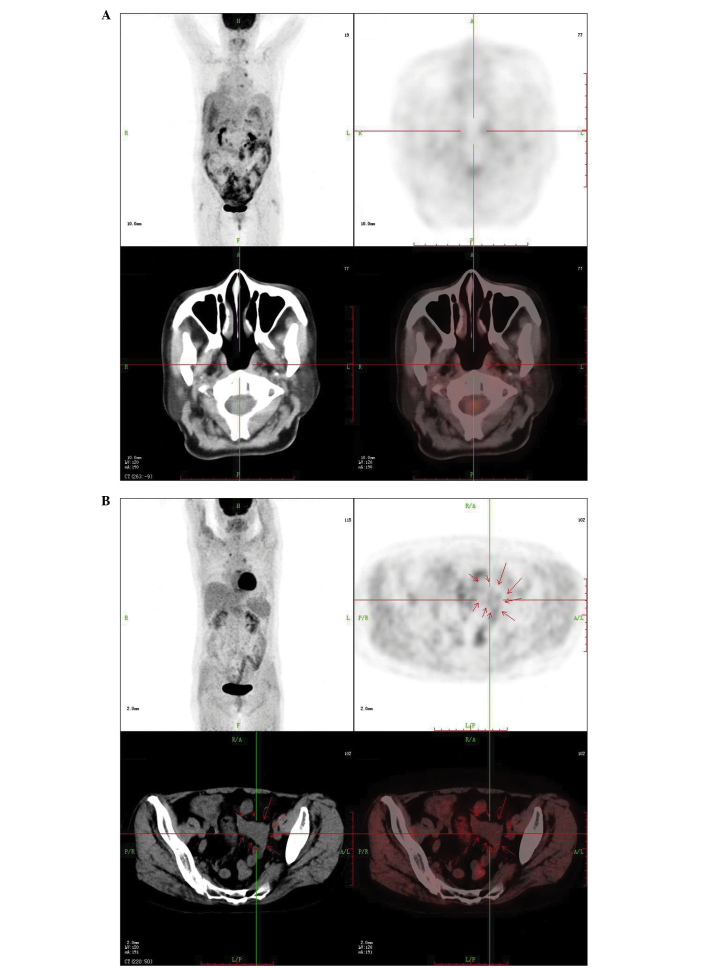
Positron emission tomography/computed tomography of (A) various regions prior to treatment and (B) normalizing ^18^F-fluorodeoxyglucose uptake in the para-aortic lymph nodes of the patient following radiotherapy treatment.

**Figure 3:**
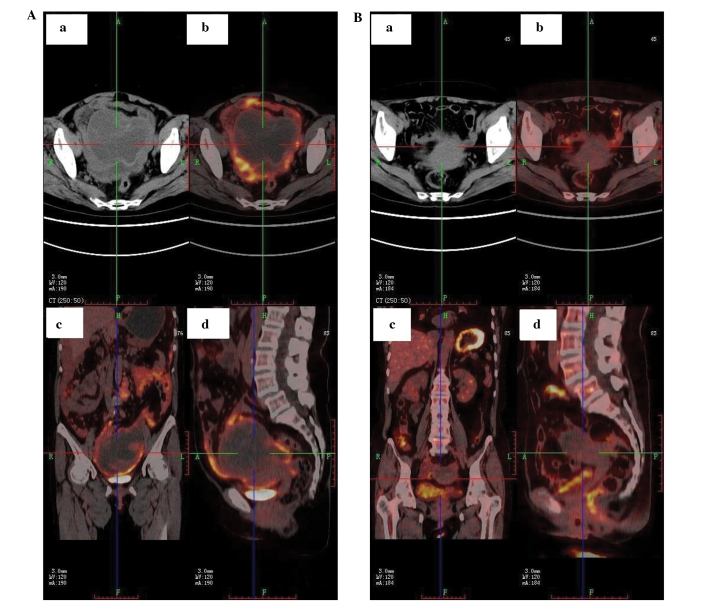
(A) Imaging of the patient prior to treatment. (Aa) Transverse CT and (Ab) transverse, (Ac) coronary and (Ad) sagittal PET scans of the patient, revealing the presence of metastatic cancer. (B) Imaging of the patient following treatment. (Ba) Transverse CT and (Bb) transeverse, (Bc) coronary and (Bd) sagittal PET scans of the patient, showing the disappearance of the tumor in the pelvic cavity. CT, computed tomography; PET, positron emission tomography.

This study presents the case of a 68-year-old female who underwent biological intensity-modulated radiotherapy (BIMRT) and neoadjuvant chemotherapy for multiple peritoneal metastases of ovarian cancer [International Federation of Gynecology and Obstetrics (FIGO) stage IIIc] (14) on February 2, 2012, at the PLA 323 Hospital. The patient presented with urination and defecation difficulties, and felt severe pain at multiple abdominal sites. The PET/CT examination indicated an ovarian cystic or solid package, frequent abdominal and retroperitoneal lymph node enlargement and abdominal cavity effusion (Fig. 1A and 2A). Transverse, coronary and sagittal PET/CT scans of the patient for peritoneal metastases prior to treatment revealed the presence of metastatic cancer (Fig. 3A).

**Figure 4:**
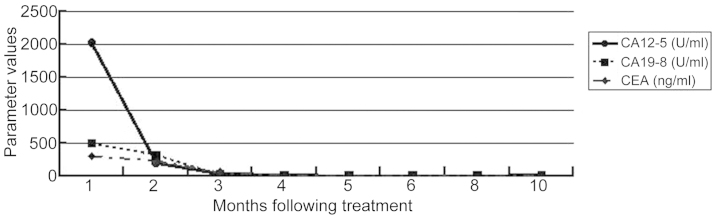
Tumor marker detection. The CA-125 level declined sharply and the CA19-9 and CEA levels declined gradually following radiotherapy treatment. CA, cancer antigen; CEA, carcinoembryonic antigen.

Following BIMRT, two cycles of neoadjuvant combination chemotherapy (180 mg Taxol and 100 mg cisplatin) were administered. In the Department of Oncology, radiation treatment was administered at a total dose of 48 Gy, as single 4.0-Gy doses 12 times. A total of 100 mg cisplatin was initially administered via peritoneal perfusion, with two cycles of chemotherapy. One chemotherapy cycle involved Taxol (180 mg/m^2^) dissolved in 500 ml intravenous saline, administered intravenously over 3 hours, following a 1-hour interval, 100 mg/m^2^ cisplatin was injected. In total, two cycles, each lasting 21 days were completed. The severe pain previously experienced by the patient, particularly the abdominal pain, was alleviated by symptomatic treatment. The tumor shrank and the patient’s condition was stabilized (Fig. 1B). Furthermore, PET/CT images following treatment showed normalizing ^18^F-fluorodeoxyglucose uptake in the para-aortic lymph nodes (Figs. 2B and 3B). Following radiotherapy treatment, the CA-125 tumor marker level declined sharply, while the CA19-9 and CEA levels declined gradually. All the tumor marker levels eventually returned to within the normal ranges (Fig. 4).

At the time of writing this manuscript, the patient is well and without relapse.

## Discussion

Ovarian cancer is one of the most commonly occurring gynecological malignancies Patients with ovarian carcinoma have a poor prognosis, as in the majority, the diagnosis is made at an advanced stage. Recurrence occurs frequently, particularly in the initial two years after first-line therapy. There have previously been no studies on the use of modern radiotherapy techniques, such as BIMRT, in ovarian cancer patients with multiple peritoneal metastases in whom surgery is not suitable. The majority of studies have reported the use of initial surgery or chemotherapy, followed by subsequent radiotherapy, for the treatment of ovarian cancer (15,16). However, the clinical outcome remains unsatisfactory (17).

The current standard treatment of maximal cytoreductive surgery and adjuvant combination carboplatin and taxane chemotherapy is aggressive, however, the prognosis for patients with an advanced disease stage remains poor. The median time to recurrence is less than two years, and the predominance of recurrence occurs intraperitoneally. The five-year survival rate for FIGO stage IIIc disease is 20–25% (18–20). According to the present results, it is reasonable to guide treatment based on the BIMRT for ovarian cancer patients with multiple peritoneal metastases in whom surgery is not suitable. Rochet *et al* (21) showed the clinical feasibility of intensity-modulated whole abdominal radiotherapy in combination with modern chemotherapy and surgery. The technique provides coverage of the entire peritoneal cavity, including frequent sites of abdominal recurrence, such as the diaphragm and liver capsule, and also the pelvic and para-aortic lymph node regions. The treatment effectively spares the kidneys, liver and bone marrow, and is subject to high compliance by patients.

In conclusion, the present study indicates that when adjuvant combination chemotherapy (Taxol and cisplatin) is available, modern radiotherapy techniques, such as BIMRT, may be considered as a beneficial treatment option for ovarian cancer patients with multiple peritoneal metastases in whom surgery is not suitable.
